# The pump as a turbine: A review on performance prediction, performance improvement, and economic analysis

**DOI:** 10.1016/j.heliyon.2024.e26084

**Published:** 2024-02-13

**Authors:** Abdulbasit Nasir, Edessa Dribssa, Misrak Girma

**Affiliations:** a Department of Mechanical Engineering, College of Engineering, Addis Ababa Science and Technology University, P.O. Box 16417, Addis Ababa, Ethiopia; b Department of Mechanical Engineering, Faculty of Manufacturing, Institute of Technology, Hawassa University, Hawassa, Ethiopia; c School of Mechanical and Industrial Engineering, Addis Ababa Institute of Technology, Addis Ababa University, P.O. Box 385, Addis Ababa, Ethiopia; d Sustainable Energy Center of Excellence, Addis Ababa Science and Technology University, P.O. Box 16417, Addis Ababa, Ethiopia

**Keywords:** Case study, Economic analysis, Historical development, Performance analysis, Performance improvement, Pump as turbine

## Abstract

Pumps as Turbines (PATs) are known for their ability to replace conventional turbines in Pico/micro-hydropower plants. However, selecting a suitable pump and estimating its performance in reverse mode is a challenging task. The overall efficiency of PATs is also lower when compared to purpose-made turbines. Numerous attempts have been made to analyze the performance of PAT in power-generating applications, and many scholars have presented their research on performance improvement and economic analysis. In this paper, a detailed review is conducted to show the historical development and current status of PAT technology. The review also presents the findings of performance prediction, performance improvement techniques, and economic analysis. The results of the literature survey demonstrate that the choice of an appropriate pump for a specific application significantly affects the performance of the PAT system. Among the various options, the Shear Stress Transport (SST) k-ω and standard k-ε turbulence models are widely used for simulating the pump as a turbine. Blade grooving and blade tip rounding are recognized as the most promising techniques for improving efficiency, with gains of 4.91% and 4.00%, respectively. Except for impeller diameter trimming, blade modification techniques proposed by various scholars have a significant impact on the efficiency of PATs; however, further research is needed to investigate the economic advantages of impeller modification.

## 1 Introduction

The global focus on meeting the energy demand has drawn attention to the state of electricity access, particularly in developing countries where it remains at a significantly lower level [1]. Governments and scholars in the field emphasize the development of environmentally friendly energy sources. Among all renewable resources, Pico and micro-hydropower (1–500 kW) are considered the most feasible, decentralized, environmentally suitable, and sustainable energy sources [[2], [3], [4], [5], [6], [7], [8], [9], [10], [11]]. In contrast to large hydropower projects, Pico/micro-hydro power resources occupy a relatively small area and do not result in significant displacement of communities from their original locations. Therefore, these energy sources are considered highly suitable in many regions of the world without giving rise to controversial issues between nations. However, using conventional turbines for Pico/micro-hydropower projects is not economically viable in developing countries [[12], [13], [14]]. As a low-cost alternative, pumps can be used in reverse mode to replace purpose-made turbines.

Hydraulic pumps serve several purposes, including liquid transportation, agricultural irrigation, and domestic and industrial applications [15,16]. Additionally, when operating in reverse mode, pumps can be utilized to generate electrical power. PAT technology finds application in various fields, including Pico/micro-hydropower projects [[17], [18], [19], [20], [21], [22], [23], [24], [25]], water supply/distribution systems [[26], [27], [28], [29]], reverse osmosis systems [29,30], pressure-reducing systems [31,32], and power recovery in irrigation and industrial piping networks [33,34].

PATs offer numerous advantages over conventional turbines, such as lower investment and maintenance costs, easy availability, and suitability for a wide range of hydraulic conditions [35,36]. From an economic perspective, it is commonly observed that PATs used in Pico/micro-hydropower projects have investment payback periods of around 2 years [[37], [38], [39]], which is significantly lower compared to purpose-made turbines.

The unavailability of pump characteristics for reverse operation in manufacturer catalogs [15,37] and the generally lower efficiency of PATs [[40], [41], [42]] are the two primary limitations when considering the use of pumps as turbines.

To highlight pump selection, performance prediction, and feasibility of PAT for power-generating applications different scholars have presented review papers. The output of performance prediction and performance improvement of PAT was presented [[43], [44], [45], [46]]. However, the review papers [16,40,47,48] exclude many performance improvement techniques. Ismail et al. [49] considered only centrifugal pumps but not axial pumps operating in turbine mode for power generation. Additional Aidhen et al. [20] and Vasanthakumar et al. [50] are also reported in the area while economic analysis of the PAT for electricity generation is not included.

This review examines the technical and economic aspects of PAT, considering original published articles. It extensively discusses various methodologies proposed for selecting PAT and presents three performance prediction approaches: theoretical/empirical, numerical, and experimental methods. The analysis aims to provide a comprehensive understanding of current advancements in PAT performance for power generation. Furthermore, it explores different techniques suggested by researchers worldwide to modify performance. Feasibility studies on PAT for electricity generation are also covered, along with future research directions including pump selection, performance prediction, performance improvement, and economic analysis for rural electrification purposes.

A comprehensive literature search was conducted to find out appropriate publications on PAT for power generation applications. Databases such as Google Scholar, ResearchGate, Scopus, Science Direct, Web of Science, PubMed and many more were searched without any restrictions on publication date or language. Many search terms were used, such as “PAT performance analysis,” “PAT historical development,” “Economic analysis of PAT,” “Case study,” and “PAT performance improvement.” The keywords, abstracts, titles, and main texts of the indexed articles were all searched for using these terms. Furthermore, a manual search was conducted through the reference lists of the identified literature to find additional relevant studies. The gathered articles were screened for original publications, and Microsoft Excel spreadsheets were used for data extraction. Studies that were not written in English, and contained insufficient research data or information were eliminated.

## 2 Historical development and the current status

The primary purpose of a hydraulic pump is to increase the water pressure and facilitate fluid movement from one location to another [51]. Additionally, pumps have a long history in power generation applications, although the exact origins of pumps used for such purposes are unclear [15]. D. Thoma and C. Kittredge [52] conducted tests around 1931 to analyze pump characteristics and incidentally discovered that hydraulic pumps work effectively in reverse. R. Knapp [53] presented comprehensive features of pumps based on experimental studies. Pump storage power plant models in the 50–100 MW range gained popularity in developed nations during the 1950s and 1960s [50]. Subsequently, chemical industries, water supply/distribution networks, reverse osmosis systems, pressure-reducing systems, and energy recovery in irrigation networks emerged as additional areas for implementing PAT technology [16,46]. This circumstance allowed for an efficient research period, during which standard pumps were used in turbine mode. In later years, many more performance analysis and performance improvement techniques were developed. Fernandez et al. In later years, numerous techniques for performance analysis and improvement were developed. Fernandez et al. [54] contributed to advances in electrical apparatus control technologies, torque, and rotation sense. S. Rawal and J.T. Kshirsagar [55] investigated pumps in turbine mode for water supply network applications. S. Derakhshan and A. Nourbakhsh [56] examined the field applications of axial pumps, multistage, and single impeller centrifugal pumps, comparing them with Kaplan, Pelton, and Francis turbines, respectively. A. Agostinelli and L. Shafer [57] extensively studied pumps in reverse operation and found that their efficiency remains the same as in direct mode, with higher pressure head and flow rate at the best efficiency point (BEP). Vasanthakumar et al. [50] reviewed research works in the field of PAT and discussed the selection of optimum pumps for different locations. Simão et al. [36] investigated the importance of PATs operating in parallel, revealing that parallel operation allows for a wider discharge range and reduces shock losses within the impeller. Fontanella et al. [58] proposed a performance database called Redawn, which is used for predicting PAT characteristics. Pienika et al. [59] highlighted the main features of axial pumps used as turbines. Most existing works focus on radial flow pumps as turbines [60] due to their lower cost and availability. Recent publications concentrate on studying flow features such as rotational speed [53,55], transient behavior [54,56], tip leakage flow [[61], [62], [63]], entropy production [60,64], and cavitation [65,66] in PATs. Furthermore, there are numerous research works focused on performance improvement [40,67], and a relatively small number of papers examine the economic analysis [61,62] of PATs.

## 3 Pump selection and performance prediction

Selecting an appropriate pump as a turbine is always challenging because most pump manufacturers do not provide reverse performance data for their pumps [15,16,68,[63], [69], [70]]. The performance of a PAT is highly dependent on operating conditions, such as the head and flow rate at the site [71]. Chapallaz et al. [72] introduced a chart that aids in selecting the optimal pump based on more than 80 test results. Radial flow pumps can be used as hydraulic turbines within a head range of approximately 10–150 m and a discharge range of 0.003–0.5 m^3^/s. Mixed flow PATs exhibit pressure head values ranging from 7 to 50 m and discharge values ranging from 0.002 to 0.75 m^3^/s. Multistage-radial flow pumps are more suitable for pressure heads between 50 and 1000 m and discharge rates between 0.001 and 0.03 m^3^/s. Axial flow pumps are preferable for pressure heads ranging from 2 to 7 m and volume flows between 0.04 and 10 m^3^/s. Double suction pumps are well-suited for operating conditions with a head range of 10–200 m and a flow rate of 0.2–0.7 m^3^/s. Numerous theoretical/empirical, numerical, and experimental investigations have been conducted and documented in the literature to predict the performance of pumps in the reverse direction.

### 3.1 Theoretical/empirical approaches

A mathematical model was implemented to study the performance of PAT by converting partial differential equations (PDE) into ordinary differential equations (ODE) [68]. In a micro-hydro power application, a statistical method was employed to select a suitable PAT, followed by performance analysis using numerical [38] and experimental [73] approaches. However, the study does not provide a comprehensive understanding of the relationship between site characteristics and pump hydraulic data. Yang et al. [69] introduced the entropy production theory to evaluate the irreversible losses in axial flow PATs. The most significant method for selecting a suitable pump to be used in the reverse direction is by employing conversion factors at the BEP for pressure head and flow rate [36,74]. The work conducted by Kramer et al. [75] demonstrated the technical and economic feasibility analysis of a stainless-steel pump. Conversion methods were subsequently established to predict the BEP of the pump when used as a turbine (Eq. (1)). The authors suggested that experimental studies are essential for accurately determining the complete characteristics curve.

(1) $${\eta bep,}_{T}={\eta bep}_{,P}\pm 0.02$$

The BUTU method (the pump as a turbine in Spanish), based on curve fitting of experimental data, is presented [76]. This method evaluates the performance of PAT in terms of output power (Eq. (2)), pressure head (Eq. (3)), and efficiency (Eq. (4)) as follows.

(2) $$\frac{P_{p}}{P_{t}}={2\eta }_{p}^{9.5}+0.205$$

(3) $$\frac{H_{p}}{H_{t}}={0.85\eta }_{p}^{5}+0.385$$

(4) $$\eta_{t}=\eta_{p}-0.03$$

where p and t represent the pump in direct and reverse mode.

Wang et al. [77], Yang et al. [37], Barbarelli et al. [38], Pienika et al. [59], and Venturini et al. [78] assumed the same rotational speeds for both pump direct and reverse modes. According to the infinite-blade theory, Euler's head ($H_{t}$) in pump direct and reverse modes is similar.

(5) $$H_{t}=\frac{1}{g}\left(u_{2}v_{u2}-u_{1}v_{u1}\right)$$

where $v_{u1}$ and $v_{u2}$ represent the peripheral elements of velocity at the high and low-pressure sides, respectively. The variable $u_{1}$ and $u_{2}$ denote the peripheral speeds, and g represents the gravitational acceleration. In Eq. (5), $v_{u2}$ tends to zero; therefore, Euler's head (Eq. (6)) is defined as:

(6) $$H_{P\;Euler}=\frac{u_{1}v_{u1}}{g}=H_{t\;Euler}$$

Due to the slip caused by the finite number of blades, the theoretical head (H″) for both the pump (Eq. (7)) and turbine (Eq. (8)) is given by:

(7) $$H_{p}^{\prime \prime }=\mu H_{p\;Euler}$$

(8) $$H_{t}^{\prime \prime }=H_{t\;Euler}/\lambda$$

where μ and λ are the slip factors for pump (Eq. (9)) and PAT (Eq. (10)) mode operation, respectively.

(9) $$\mu =\frac{{\Delta H}_{p}}{H_{Euler}}$$

(10) $$\lambda =\frac{{\Delta H}_{t}}{H_{Euler}}$$

Capurso et al. [79] worked on slip factor correction in a one-dimensional performance prediction model for PAT. To further evaluate the significance of accounting for the slip phenomenon at the outlet of the runner, the model was applied to another double-suction centrifugal pump. This pump was tested in both operating modes and shares similarities with the referenced machine, including a similar specific speed number, the same number of blades, and a similar outlet blade angle.

From the fundamentals of energy transfer in hydraulic turbines, the output power ($P_{t}$), theoretical head ($H_{t}$), and hydraulic efficiency ($\eta_{h}$) can be represented by Equations (11), (12), (13) [80]: In Equation (13), $H_{\text{total}}$ represents the sum of both losses and theoretical head. The conversion factor proposed by the researchers is summarized in (Table 1).

(11) $$P_{t}=\rho gQH_{t}-P_{mech}-P_{leak}$$

(12) $$H_{t}=\sigma H_{Euler}$$

(13) $$\eta_{h}=\frac{H_{t}}{H_{total}}$$

**Table 1:** Conversion factors proposed by different scholars.

Authors	q	h
Yang et al. [81]	1.55	1.99
Alatorre-frenk [76,82], Bogdanovic-Jovanovic et al. [76]	$\frac{0.85\eta_{P}^{5}+0.385}{{2\eta }_{P}^{9.5}+0.205}$	$\frac{1}{0.85\eta_{P}^{5}+0.385}$
Huang et al. [83], Yang et al. [37], Barbarelli et al. [38], and Wang [77]	$\frac{Q_{T}}{Q_{P}}$	$\frac{H_{T}}{H_{P}}$
Stepanoff [84]	$\frac{1}{\sqrt{\eta_{P}}}$	$\frac{1}{\eta_{P}}$
Sun-Sheng [39]	$1.2\eta^{-0.55}$	${1.2\eta }^{-1.1}$
Aidhen et al. [85]	–	1.56

Stefanizzi et al. [86] developed a performance prediction model for multistage centrifugal pumps used as turbines with two-phase flow. In their study, a 6-stage centrifugal pump operating as a turbine was used as a case study to develop a theoretical model. The goal was to predict the performance of a pump as a turbine operating with a two-phase flow, where the properties of the fluid are known. In this model, the fluid is assumed to have no vapor phase at the inlet section of the machine, while both the mass vapor fraction and the volume vapor fraction are increased during the expansion process.

The velocity triangle method is another theoretical approach used to analyze the performance of a PAT [37]. It involves analyzing the velocity vectors of the water at both the outlet and the inlet, and evaluating the power output and efficiency based on the resulting fluid velocities.

The performance of a 4-blade axial flow pump equipped with a 980 rpm induction generator in both pump and turbine modes was examined [59]. To gain a better understanding of the interplay between these two operations, the researchers utilized the Euler equation and velocity triangles. The findings highlight that utilizing an axial pump as a turbine instead of traditional axial turbines offers a compelling alternative for reducing investment costs.

When delving into the energy conversion characteristics of the PAT, the theoretical approach can only provide a macroscopic perspective [22]. As a result, the theoretical approach is suitable for investigating flow domains with limited parameters, but it becomes challenging to examine PATs in detail.

### 3.2 Numerical investigations

Computational Fluid Dynamics (CFD) is considered a suitable tool for determining the characteristics curve and BEP of the PAT. Bozorgi et al. [12] conducted simulations of a pump operating in the reverse direction using computational fluid NUMECA software. The study focused on low-head Pico-hydro resources. The simulations involved solving the Reynolds-averaged Navier Stokes (RANS) equations coupled with the Spalart-Allmaras turbulence model. The results showed that axial pumps can be viable alternatives to conventional turbines in low-head hydropower sites. Simão et al. [36] presented a numerical investigation of two PATs operating in parallel and single arrangements. The study highlighted the advantages of PATs operating in parallel, particularly in terms of covering a wider range of discharge compared to single-mode operations. Frosina et al. [74] predicted the performance of three pumps with specific speeds (Ns = 37.6, 20.5, and 64.0) operating as turbines at various flow rates (8–21 L/s) using numerical and experimental approaches. The results showed that the pump's performance improves as the flow rate increases from 8 to 21 L/s. It was also noted that optimizing the impeller blades is crucial for achieving better performance in power-generating applications when using pumps as turbines.

Telikani et al. [87] proposed an evolutionary Artificial Neural Network (ANN) based on a typology of the differential evolution algorithm to predict the performance curves and BEP of a PAT using information obtained from the pump's direct mode. The accuracy of this approach was assessed through experimental tests.

In the study by Miao et al. [88], a single-stage pump operating in the opposite mode was selected and simulated using 3D modeling software called Pro/Engineer. The pump had the following parameters: discharge = 12.5 m^3^/h, head = 30.7 m, rotating speed = 2900 rpm. A structured hexahedral mesh was created using ICEM. The study aimed to evaluate the variations in power across different sections of the impeller under different working conditions. The input power in different areas of the impeller blade and the energy transfer from the water to the impeller were analyzed. The impeller was divided into 6 sections, with the front and central parts identified as the significant sections where work is primarily done by the fluid. The work done by the fluid in the rear area was relatively small, indicating the need for optimization in the impeller's rear part to improve performance. Shi et al. [89] conducted an unsteady 3D simulation based on the RANS equations to analyze the turbine mode with a guide vane. The study focused on the pressure fluctuation behavior and the stability of hydraulic turbine operation. It was observed that the pressure fluctuation varied with different flow rates, with higher flow rates resulting in more significant pressure fluctuations.

Bogdanovic-Jovanovic et al. [76] analyzed the characteristics of the turbine mode and investigated improvements in the internal hydraulic performance of a PAT using the commercial code Ansys CFX. The simulation employs the 3-D incompressible RANS equations coupled with the standard k-ε turbulence model. An unstructured computational mesh is utilized, with a fixed number of 270,373 nodes and 1,116,956 elements determined after conducting the grid independence study. The overall pump characteristic is evaluated by considering variables such as net head, hydraulic output torque, discharge, and rotational speed.

Scholars have also examined both the direct mode (pump) and inverse mode (PAT) operations [90]. In the study of Kan et al. [62], the energy loss characteristics and flow dynamics of an axial-flow PAT in both pump and turbine modes are analyzed. To achieve this, a numerical simulation approach is employed, utilizing the entropy production theory. The study aims to develop an energy loss intensity model in the cylindrical coordinate system. This model quantitatively describes the spatial variation pattern of energy losses in both pump and turbine operating modes.

Yang et al. [37] presented the performance prediction of a centrifugal pump using CFD in both pump and turbine modes. To obtain more accurate results, a hexahedral structured mesh was created using ICEM-CFD. The computational domain was divided into five sections: volute, front chamber, impeller, back chamber, and inlet pipe. To validate the simulated results, the pump was manufactured and tested on an experimental test rig.

**Fig. 1:**
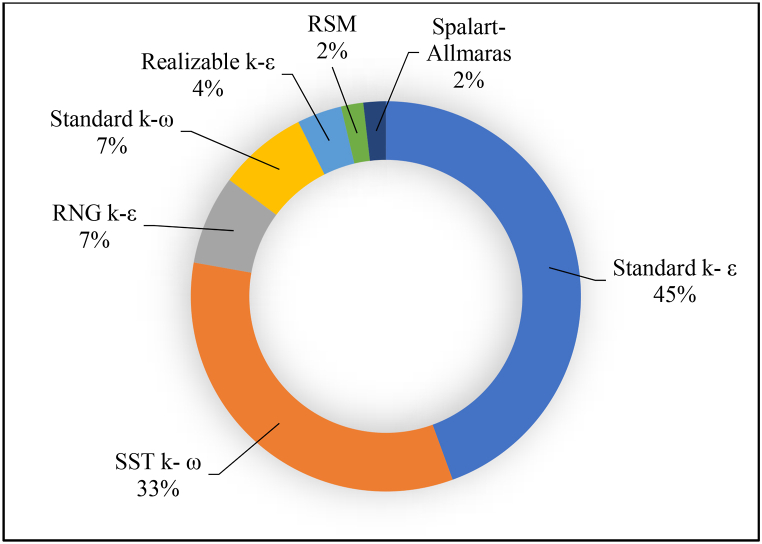
Summary of turbulence models.

Renzi et al. [91] conducted a case study that examined the installation of an axial pump operating as a turbine in a branch of the wastewater sewer in Italy, considering both technical and economic aspects. The study identified an appropriate design for the axial PAT using Ansys Workbench, and CFD simulations were conducted for the PAT design in both pump and turbine modes. The results indicate that axial flow pumps offer great potential as a viable alternative to conventional turbines. (Table 2) and (Fig. 1) provide an overview of the turbulence models applied by different researchers. Based on the literature survey, it is found that the standard k-ε and SST k-ω turbulence models are widely used, accounting for 45% and 33% respectively.

**Table 2:** Turbulence models applied by different researchers.

Turbulence model	No. of papers	Author
Standard k- ε	24	[35,68,38,53,63,69,92,71,72,83,76,[78], [79], [80], [82], [84], [85], [86], [87], [88], [89], [90], [91], [93]]
SST k- ω	18	[54,57,58,60,72,82,[93], [94], [95], [96], [97], [98], [99], [100], [101], [102], [103], [104]]
RNG k-ε	4	[69,72,77,82]
Standard k-ω	4	[72,85,93,105]
Realizable k-ε	2	[82,106]
RSM	1	[104]
Spalart-Allmaras	1	[12]

### 3.3 Experimental analysis

Testing the characteristics of a PAT through a laboratory setup is considered the most reliable approach for predicting its performance. However, it is important to consider the potential effects of measurement inaccuracies when conducting experiments. Raman et al. [107] conducted experiments on a radial flow pump with a specific speed of 15.36 to determine its reverse performance. The peak flow rate and pressure head were 8.3 l/s and 22 m, respectively, and the pump operated at a speed of 1500 rpm. Non-dimensional parameters, such as the head coefficient (ψ), discharge coefficient (φ), and power coefficient (π), were used to present the results (Equations (14), (15), (16)) [108,109].

(14) $$\Psi =\frac{gh}{n^{2}D^{2}}$$

(15) $$\varphi =\frac{Q}{nD^{3}}$$

(16) $$\pi =\frac{P}{\rho n^{3}D^{5}}$$

Ismail et al. [110] conducted an experimental design and investigation of a PAT for a micro-hydro system, evaluating its performance across various rotational speeds. The maximum flow and head achieved were 8 l/s and 14 m, respectively. The power produced, available hydraulic input power, and overall efficiency can be expressed by Equations (17), (18), (19), respectively. The tested pump exhibited a peak efficiency of 65.04% in turbine mode. However, there was a significant decline in efficiency at off-design points.

(17) $$P_{mechanical}=\tau \times \omega \;\left(Watt\right)=\frac{2\pi N\tau }{60}\left(Watt\right)$$

(18) $$P_{hydraulic}=\rho gQH\;\left(Watt\right)$$

(19) $$\eta =\frac{P_{mechanical}}{P_{hydraulic}}\times 100\;\left(\%\right)$$

Lin et al. [96] conducted tests on a low-specific speed pump operating in reverse mode using an experimental test rig. The voltage, current, flow rate, and pressure head of the PAT were measured. The results showed a good agreement between the model and experimental results under all tested conditions. In the study by Rossi et al. [34], experimental tests were performed to analyze the performances of both modes. Torque, mechanical power, and efficiency were evaluated under different load conditions. The findings indicate that a peak efficiency of 76% can be achieved in both the direct and reverse modes when operating at a specific speed of 0.57. In comparison to other related studies (e.g., Refs. [107,110]), the disparity between the numerical values of flow rate and head of the pump in both forward and reverse modes is large.

Delgado et al. [111] conducted experiments to investigate the variable speed operation of a centrifugal pump as a turbine. They tested three single-stage centrifugal pumps with specific speed values of 23.1, 41.0, and 67.3. The results demonstrate that operating the machines at variable speeds expands the discharge-specific energy operating range and improves efficiency. Additionally, the extended operation revealed that these hydraulic machines do not exhibit an instability region near the runaway conditions. The researchers recommended further research to develop a control algorithm that can effectively manage the discharge or downstream pressure in PAT systems.

Aidhen et al. [85] suggested that care should be exercised when using a pump as a turbine in small hydropower plants. It is important to carefully control and maintain the output of the PAT within the appropriate range for the shaft, bearings, and couplings. Generally, both numerical and experimental results have shown that pumps can be successfully used as hydraulic turbines without encountering mechanical difficulties. However, to achieve optimal performance, some parameters of the pump must be modified before it is used as a turbine [69,76]. The error (Eq. (20)) between the simulation and experimental results for different PAT parameters is presented in (Table 3). The turbulence models used in the studies are also specified, and it is observed that the standard k-ε model exhibits both maximum and minimum deviations (error). This indicates that the accuracy of numerical simulations is influenced not only by the choice of turbulence model but also by other factors such as software type, grid size, physics setup, and more.

(20) $$Error=\frac{CFD\;value-Experimental\;value}{Experimental\;value}\times 100$$

**Table 3:** Comparison of simulated and tested outputs at the BEP.

Author	Parameters	Turbulence model	Numerical value	Experimental value	Absolute error (%) ≈
Bozorgi et al. [12]	ψ	Spalart-Allmaras	2.54	2.59	1.93
	π		0.67	0.69	2.90
	η		62.23	61.05	1.93
Bogdanovic-Jovanovic et al. [76]	H	Standard k- ε	14.95	14.78	1.15
Yang et al. [37]	H	Standard k- ε	33.40	32.40	3.09
	P		4.72	4.56	3.51
	η		60.31	59.98	0.55
Sun-Sheng et al. [81]	H	Standard k- ε	33.71	32.40	4.04
	P		4.98	4.56	9.21
	η		63.03	59.98	5.09
Sun-Sheng et al. [39]	H	Standard k- ε	40.69	41.04	0.85
	Q		95.00	96.99	2.05
	P		6.92	6.78	2.06
	η		65.77	62.53	5.18
Miao et al. [88]	H	RNG k-ε	47.86	46.22	3.55
	η		62.43	58.60	6.54
Shi et al. [89]	H	Standard k- ε	127.71	123.07	3.77
	η		83.02	78.71	5.48
Shi et al. [112]	H	Standard k-ω	165.50	157.84	4.85
	P		154.10	144.26	6.82
	η		76.28	75.35	1.23
Shi et al. [94]	H	Standard k- ε	44.84	46.00	2.52
	η		73.83	70.85	4.21

The output of the CFD simulation showed excellent agreement with the experimentally tested results, with a deviation of less than 10%. In many cases, the numerically predicted values demonstrated high accuracy and matched well with the experimental data. As a result, computational fluid dynamics is considered a suitable approach for analyzing the performance of pumps operating in reverse mode and determining the optimal working point.

### 3.4 Case studies

This subsection provides a summary of research papers that have examined the installation of PAT systems in real water distribution networks. These studies focus on utilizing PATs as a replacement for pressure regulation valves and recovering a substantial amount of energy.

Balacco et al. [113] developed a tool that can assist in selecting the pump as a turbine based on specific characteristics of water distribution networks (WDNs). This tool is designed to apply to any WDN and machine catalog, and it can simulate water demand patterns when flow rate data at the installation site is unavailable. The researchers introduced an algorithm that has been automated in a computer tool called PaT-ID. The effectiveness of the algorithm was evaluated by testing it on the WDNs of two towns in the Apulia region of Southern Italy as case studies. The results demonstrate the code's excellent potential in aiding decision-makers and reducing time-to-market. However, it is important to note that this approach does not calculate energy production or include an economic analysis of the PAT.

Alberizzi et al. [114] examined a practical scenario in Laives, a small city in the South Tyrol region of Italy, to demonstrate the benefits of speed control in PAT. To establish a correlation between the pump and turbine modes, the researchers determined the specific speed and specific diameter of the machine while operating in pumping mode, and compared it to the specific speed and specific diameter of the machine while operating in turbine mode.

Rossi et al. [28] conducted a study to explore the potential of PAT for energy recovery and pressure regulation. The researchers specifically examined the Egna municipality Water WDN located in Northern Italy. The selection process for PATs involved analyzing the available flow rates and heads within the network. To evaluate the BEP parameters of each PAT, analytical relationships based on specific speed and specific diameter were established through experimental tests conducted on multiple PATs.

Marini et al. [27] introduced a novel approach to the selection of pumps as turbines. The researchers applied the proposed methodology to a practical network to demonstrate the advantages of replacing a pressure-reducing valve with a PAT in a real water distribution network district located in Benevento, Italy.

Filannino et al. [115] conducted an experimental test to explore the performance of a PAT within a potential layout integrated into a WDN. The machine used in the study was selected using a specific procedure to ensure its BEP aligns with the average flow rate of the daily flow pattern of an Italian WDN located in Southern Italy. PAT tests were conducted at a constant speed, employing a typical hydraulic control scheme and incorporating a bypass. The results demonstrate the feasibility of recovering a significant amount of hydraulic energy through the implementation of a bypass hydraulic control scheme.

Fontana et al. [116] examined a WDN in the city of Naples to evaluate the potential benefits of energy recovery instead of dissipating excess pressure. Their analysis revealed that implementing energy recovery measures could result in substantial energy savings and a significant reduction in water loss. Additionally, the researchers conducted a preliminary economic analysis for the installation of PAT, which demonstrated attractive profit potential and a reasonable capital payback period.

An innovative booster set featuring two centrifugal submersible borehole pumps operating as a turbine was introduced in a study by Carravetta et al. [35]. The research also presented an optimal hydraulic regulation scheme for the plant, aiming to achieve a balance between low installation costs and simplified plant management. To conduct the study, a branch of an actual WDN situated in the village of Granville in Normandie, France, was selected as a case study. The findings of this research can provide valuable insights for network managers and technicians seeking to enhance energy efficiency and implement energy recovery in their networks.

Morani et al. [117] conducted a study using a real water network located in Ireland, approximately 100 km away from the capital city, Dublin. The research focused on comparing direct pumping and indirect pumping methods, specifically evaluating the efficiency of the system when equipped with energy recovery devices such as a PAT. The case study findings suggest that direct pumping can be more efficient than indirect pumping.

## 4 Performance improvement

The efficiency of PATs is typically lower than that of Francis turbines [118], which are commonly used for hydroelectric power generation. Additionally, PATs often have lower efficiency compared to cross-flow turbines [119], which are frequently utilized in micro-hydropower plants in developing regions. Generally, PATs exhibited lower performance compared to purpose-made turbines. The main reason for this is that some components specially impeller blades are not suitable to operate in the reverse direction [75,88]. Therefore, when using the pump as a turbine, it is necessary to properly enhance the impeller blades to achieve maximum performance.

The performance of the PAT can be enhanced through various methods, including blade profile optimization [95], blade tip rounding [13,71,120], impeller diameter trimming [13], adjusting the number of blades [112], implementing splitter blades [39,[121], [122], [123]], blade grooving [102], and modifying blade thickness [80].

Optimization of a single-stage centrifugal pump operating in turbine mode was carried out in a numerical study [95]. In this work, nine coordinate values were considered as control points. The efficiency of the modified PAT increased by 2.91%. Although there was a slight increase in hydraulic loss in the outlet pipe and volute; overall, the total loss decreased.

The effects of impeller blade rounding on the performance of the PAT were investigated in studies [13,120]. The efficiency of the PAT increased by up to 4% at the rated speed when the original impeller was used in different operating sections. In the research conducted by Derakhshan et al. [71], the impact of blade rounding on the performance of the PAT, with a specific speed of 23.5 as a turbine, was presented. A gradient-based optimization approach, in conjunction with a 3D Navier-Stokes flow solver, was employed to modify the blade shape and achieve higher efficiency in turbine mode.

Yang et al. [81] conducted numerical simulations and experimental examinations on an axial flow PAT with different impeller diameters of 255 mm, 235 mm, and 215 mm. The study found that the efficiency and flow rate at the BEP decreased by 2.17% and 20 m^3^/h, respectively, as the impeller diameter decreased from 255 mm to 215 mm. A review presented by Liu et al. [40] revealed that impeller trimming negatively affects performance; however, it is important to adjust the best efficiency point. In the experimental investigation conducted by Jain et al. [13], the influence of impeller diameter (250 mm, 225 mm, and 200 mm) on the performance of the PAT was studied. The study found that the maximum efficiency was achieved at a diameter of 225 mm.

The influence of the number of blades (10, 11, 12, and 13) on the performance of the PAT was analyzed both numerically and experimentally [112]. Numerical calculations were performed using ANSYS CFX, and the results were validated using experimental data. It was found that when the number of blades was 10, the PAT exhibited the highest peak efficiency and the flow became more stable. Similarly, Ismail et al. [103] investigated the impact of blade numbers (5, 6, 7, and 8) on the PAT's performance. The study revealed that the highest efficiency was achieved when the PAT had 7 blades.

The effects of splitter blades on the performance of PAT were investigated both numerically and experimentally by Yang et al. [39]. The study focused on a PAT with a rotational speed of 1500 rpm. It was found that after the implementation of splitter blades, the efficiency was increased by 3.42%.

In the numerical research presented by Yang et al. [80], the effect of blade thickness on the performance of the pump as a turbine was investigated. The study revealed that reducing the blade thickness resulted in a decrease in total hydraulic loss and an increase in efficiency. Furthermore, the research highlighted the influence of blade inlet width and blade angles on the performance of the pump as a turbine.

The effect of blade inlet angle, which is an important parameter for guiding the flow appropriately [102], on the performance of the PAT was investigated. The study determined that the suitable range of blade inlet angle is 25°–35° [95]. CFD was employed to evaluate blade inlet angles of 20°, 25°, 30°, and 35° [81]. The results showed that as the blade angle increased from 20° to 35°, the flow at the BEP improved by 2.50 m^3^/h, and the maximum efficiency was achieved at a blade inlet angle of 30°.

Zhao et al. [97] conducted a study to investigate the performance enhancement of an axial-flow pump operating in turbine mode with the implementation of inlet guide vanes (IGVs). The researchers redesigned and installed adjustable IGVs with five different angles in front of the impeller of the axial pump, aiming to expand the operating range for high efficiency in PAT mode. The obtained results indicate that the adjustable IGVs significantly influence the performance of the axial pump, particularly by expanding the high-efficiency regime in PAT mode. In a related study, Qian et al. [124] performed a performance evaluation of an axial-flow pump operating in turbine mode, utilizing adjustable guide vanes. The motivation behind using the PAT instead of a conventional turbine was to reduce the initial capital cost and payback period.

Bayatloo et al. [41] conducted an experimental study on performance improvement of a pump operating as a turbine for energy recovery considering the effects of polymer additives. The study focuses on investigating the impact of polymer additives, specifically drag-reducing agents, on the performance of a centrifugal pump operating in turbine mode. The results of the study demonstrate that the utilization of working fluids with viscoelastic properties, containing additive polymers, leads to significant performance improvements for both reverse and direct pumps.

A new technique called blade grooving was introduced by Nasir et al. [102] to examine its impact on the performance of a single-stage pump operating as a turbine. In their numerical study, they achieved a 4.9 percent improvement in efficiency. The effects of various modification techniques proposed by different researchers for different pumps are summarized in (Table 4).

**Table 4:** Results of PAT modification techniques.

Author	Methods	Efficiency improvement (%)
Sun-Sheng et al. [39]	Splitter blades	3.42
Shi et al. [112]	Number of blades	1.75
Miao et al. [95]	Optimization	2.91
Derakhshan et al. [71]	Blade rounding	4.00
	Optimization	2.75
Singh and Nestmann [120]	Blade rounding	2.00
Yang et al. [80]	Blade thickness	1.40
Xia Shi et al. [94]	Guide vane numbers	2.00
Nasir et al. [102]	Blade grooving	4.91
Yu et al. [122]	Splitter blades	1.30

Among the various techniques explored by scholars to enhance the performance of the PAT, two promising techniques have emerged: blade grooving and rounding the impeller at the inlet. These techniques have shown significant efficiency improvements, with blade grooving achieving an efficiency improvement of 4.91% and impeller rounding at the inlet resulting in a 4.00% efficiency improvement.

## 5 Economic analysis

The sustainability of Pico/micro-hydro projects for rural electrification can be achieved by employing a sustainability assessment model that encompasses social, economic, environmental, and technical aspects [8,110]. In these plants, the cost of electro-mechanical components is relatively higher compared to large hydro systems, accounting for approximately 35–40% of the total project cost. However, this cost can be reduced by utilizing PAT technology instead of purpose-made turbines [19,125].

Relatively, a limited number of studies are focused on the economic analysis of PAT for power-generating applications. In their study, M. De Marchis et al. [68] investigated the economic advantages of utilizing PAT technology in the WDN. The objective was to assess the economic benefits of energy recovery through PATs from the fluid line, instead of conventional devices. The analysis was conducted using a mathematical model and has been applied to a small town close to Palermo city (Sicily), called Misilmeri. The computed compound payback time achieved values of about 2 years or 12 years as a function of the PAT position, clearly showing that, in some cases, the investment is economically unfeasible.

Motwani et al. [19] conducted a cost evaluation comparing the PAT and Francis turbine. The comparison considered aspects such as overall efficiency, initial cost, life cycle cost analysis, and cost of electricity generation. The electricity generation cost was found to be 0.059 USD/kW for the Francis turbine and 0.012 USD/kW for the PAT.

De Marchis et al. [126] proposed the numerical model which has been applied to the WDNs of Palermo city (Sicily). To identify the most suitable solution in terms of energy power production without compromising the hydraulic performance of the network in terms of service delivery. The authors used a cost of 2000 €/kW for both the PAT and generator. The result showed that a really attractive capital payback period was achieved when the PATs were installed in the pipes located close to the water supply node.

In the research conducted by Chacón et al. [127], an advanced statistical methodology is developed to determine the power available for energy recovery through radial PATs in on-demand irrigation networks found in southern Spain. Then, a cost model was employed that estimated the cost per kW of PATs with induction generators based on the number of magnetic poles. The results of these studies collectively demonstrate that using PATs for power-generating units is cost-effective, often representing just 10% of the cost of purpose-made turbines.

Different authors have proposed various methods for estimating the cost of the PAT and generator. Ramos et al. [128] suggested a cost range of 200–400 €/kW for the PAT, specifically for nominal power lower than 40 kW. Carravetta et al. [129] planned a cost of 230 €/kW for the turbine's nominal power and 115 €/kW for the generator.

## 6 Conclusions and prospects

In various sections of this review paper, it has been recognized that extensive investigations have been conducted on the performance analysis, performance improvement, and economic analysis of the pump operating in reverse mode. Real case studies are also considered by various researchers to justify their proposed methodology.

According to our analysis, the application of PATs proves to be both technically and economically appealing alternatives for Pico/micro-hydropower applications. PAT can efficiently utilize various power sources such as natural streams, irrigation pipelines, wastewater sewers, and industrial WDNs. To address the limitations associated with PAT, it is crucial to select an appropriate pump and strategically determine the PAT's location within the piping system. The initial performance of the PAT can be enhanced by controlling the fundamental design parameters. This involves making adjustments such as changing the number of blades, application of splitter blades, decreasing blade thickness, blade profile optimization, blade tip rounding, adjusting blade angles, and blade grooving. Among the various modification techniques discussed in the literature, blade grooving and blade tip rounding have emerged as the most promising methods with efficiency improvement of 4.91% and 4.00% respectively. However, impeller diameter trimming has a negative impact on the performance of PAT. The economic analysis demonstrates that the utilization of PAT is economically viable, as long as the available stream flow surpasses the design flow rate. With the aid of the PAT position, an attractive payback period of about a year and 8 months was reported.

Additional research is needed to establish generic mathematical correlations between site-specific hydraulic data and pump performance. Analyzing the economic feasibility of PAT with impeller modifications is also a promising area for future research. Furthermore, conducting comparative studies on various turbulence models to determine the most effective one is essential. A comprehensive investigation into the choice of material for the impeller is crucial, particularly when considering the reduction of blade thickness and application of blade grooving.

## Data availability statement

Data will be made available on request.

## Additional information

No additional information is available for this paper.

## CRediT authorship contribution statement

**Abdulbasit Nasir:** Writing – review & editing, Writing – original draft, Data curation, Conceptualization. **Edessa Dribssa:** Writing – review & editing, Writing – original draft, Supervision. **Misrak Girma:** Writing – review & editing, Writing – original draft, Visualization, Funding acquisition.

## Declaration of competing interest

The authors declare that they have no known competing financial interests or personal relationships that could have appeared to influence the work reported in this paper.

## Abbreviations and acronyms

BEP: Best Efficiency Point
C_H_: Head conversion factor
C_Q_: Flow rate conversion factor
CFD: Computational Fluid Dynamics
H: Head
k: Turbulent kinetic energy
n: Rotational speed
N_s_: Specific speed
PAT: Pump as turbine
Q: Flow rate
RANS: Reynolds-averaged Navier Stokes
SST: Shear Stress Transport
WDN: Water Distribution Network
η: Efficiency
ρ: Density
ε: Dissipation
ω: Specific dissipation rate
φ: Discharge number
ψ: Head number
π: Power number
